# Investigating Influence Factors on Traffic Safety Based on a Hybrid Approach: Taking Pedestrians as an Example

**DOI:** 10.3390/s24237720

**Published:** 2024-12-03

**Authors:** Yue Li, Yuanyuan Shi, Huiyuan Xiong, Feng Jian, Xinxin Yu, Shuo Sun, Yunlong Meng

**Affiliations:** Transport Planning and Research Institute, Ministry of Transport, Beijing 100028, China; liyue86@tpri.org.cn (Y.L.); shiyy@tpri.org.cn (Y.S.); xionghuiyuan@tpri.org.cn (H.X.); jianfeng@tpri.org.cn (F.J.); yuxx@tpri.org.cn (X.Y.); mengyunlong@tpri.org.cn (Y.M.)

**Keywords:** pedestrian traffic safety, intelligent transportation systems, sensor-based data, pedestrian–vehicle crashes, hybrid approach

## Abstract

Road traffic safety is an essential component of public safety and a globally significant issue. Pedestrians, as crucial participants in traffic activities, have always been a primary focus with regard to traffic safety. In the context of the rapid advancement of intelligent transportation systems (ITS), it is crucial to explore effective strategies for preventing pedestrian fatalities in pedestrian–vehicle crashes. This paper aims to investigate the factors that influence pedestrian injury severity based on pedestrian-involved crash data collected from several sensor-based sources. To achieve this, a hybrid approach of a random parameters logit model and random forest based on the SHAP method is proposed. Specifically, the random parameters logit model is utilized to uncover significant factors and the random variability of parameters, while the random forest based on SHAP is employed to identify important influencing factors and feature contributions. The results indicate that the hybrid approach can not only verify itself but also complement more conclusions. Eight significant influencing factors were identified, with seven of the factors identified as important by the random forest analysis. However, it was found that the factors “Workday or not” (Not), “Signal control mode” (No signal and Other security facilities), and “Road safety attribute” (Normal Road) are not considered significant. It is important to note that focusing solely on either significant or important factors may lead to overlooking certain conclusions. The proposed strategies for ITS have the potential to significantly improve pedestrian safety levels.

## 1. Introduction

With the rapid urbanization in China, the urban population continues to grow. This has led to an increase in various types of traffic participants, which has further intensified the demand for urban transportation supply. However, with limited transportation resources, conflicts and issues among different traffic participants have become increasingly prominent, significantly impacting urban traffic safety. Walking, the most fundamental form of transportation, is increasingly acknowledged as an important and sustainable mode of transportation. However, it is often linked to negative public health outcomes in pedestrian–vehicle crashes. As a result, pedestrian safety has been a key focus in road safety research, given that pedestrians are more vulnerable and more exposed compared with car occupants [1]. Pedestrian–vehicle crashes present significant challenges to pedestrian safety, and pedestrian-involved fatal crashes account for approximately 17% of all road user deaths by WHO Region [2]. Intelligent transportation system provides important support for road safety data acquisition, analysis, and visualization to help prevent pedestrian fatalities in pedestrian–vehicle crashes. Currently, numerous experts and scholars are dedicated to studying pedestrian injury severity in pedestrian–vehicle crashes [3,4].

Previous studies have categorized influencing factors on pedestrian injury severity in pedestrian–vehicle crashes into four main groups: pedestrian attributes; vehicle and driver attributes; infrastructure attributes; and environmental and temporal attributes. For example, Li et al. [5] analyzed pedestrian crash injury severity based on 3 different category factors, including driver-related factors, pedestrian-related factors, and environmental factors. Munira, Sener, and Dai [6] examined pedestrian crash severity at signalized intersections using various variables, including traffic characteristics, road geometry, built environment features, and pedestrian exposure volume. Qiu and Fan [7] explored pedestrian injury severities at intersection and nonintersection locations, applying influencing factors to driver, pedestrian, vehicle, road, and environmental and temporal characteristics.

Most studies on pedestrian-involved crashes rely on sensor-based data. Generally, when a crash occurs, the police arrive at the scene to manage the situation and gather relevant crash data. In the investigation of crash scenes and crash investigations, a large amount of crash data needs to be applied with advanced equipment and technology. For example, GPS detectors are used to determine the location of the crash. Laser range finders are applied to gather information about the infrastructure. Laser scanning devices are used to collect environmental data from the crash scene. In addition, other source data are added to support research related to crash causation analysis and causal inference. Relevant time information is needed to supplement based on weather databases and holiday databases, and relevant location information is added according to geographic information systems.

In terms of model application, the random parameter logit model (RPL) is commonly used in these studies due to the complex mechanism of injury severity involving numerous influencing factors that may be unobservable to researchers. These unobserved factors can alter the effects of observed factors on injury severity, constituting unobserved heterogeneity [8,9,10]. However, the sole application of the random parameter model can only reveal the significance and heterogeneity of factors. It is challenging to uncover other relationships between factors using this model alone. In this context, many experts have proposed hybrid approaches. For instance, Chiou et al. [11] introduced a hybrid approach combining a genetic mining rule model and a mixed logit model to investigate crucial hazardous conditions that may result in severe injury in single-vehicle crashes. Sun et al. [12] proposed a hybrid method combining RPL models and Bayesian networks to study the influencing factors affecting the injury severity of pedestrian, cyclist, and motorcyclist-involved crashes. Tamakloe et al. [13] presented a hybrid approach combining binary logit regression and association rule mining. Sun et al. [14] proposed a mixed method combining random forest (RF) and RPL models to identify important factors affecting crash injury severity.

In addition, the importance of factors is crucial for injury severity, as it can inform policy prioritization [15]. For instance, Panicker and Ramadurai [16] used RF and Conditional Inference Forest to model the injury severities of two-wheeler crashes and explore the crucial factors. Jamal et al. [17,18] compared the performance of XGBoost, logistic regression, RF, and decision tree methods to capture risky factors of traffic crashes. Sharafeldin et al. [19] employed RF to capture the importance of significant factors impacting crash injury severities.

Therefore, in order to avoid the limitations of using a single method and to explore complementary results between data-driven methods and statistical models, this paper also employs a hybrid method to study pedestrian injury severity in pedestrian–vehicle crashes, in which the random parameter logit model (RPL) is applied to explore the significant factors and capture the unobserved heterogeneity, random forest based on SHAP (RF-SHAP) is applied to find out the important factors and discuss the feature contribution of the important factors. The main contributions are as follows: (1) identifying the significance and importance of influencing factors and the unobserved heterogeneity based on a hybrid approach between statistical models and machine learning approaches; (2) utilizing the RPL model to uncover the significance and heterogeneity of factors; (3) employing the RF-SHAP to explore the importance and feature contribution of the factors; and (4) making a hybrid approach attempt between statistical models and data-driven methods to study pedestrian injury severity in pedestrian–vehicle crashes.

## 2. Data and Description

This study applies pedestrian crash data from Beijing using multi-sensor detection methods provided by the police in conjunction with various other data sources. This study applies pedestrian-involved crash data in Beijing using sensor-based data. After filtering out records with incomplete or missing information, a dataset of 756 crash incidents was finalized for descriptive statistical analysis and subsequent model development. Among these, there are 401 pedestrian fatal crashes and 355 pedestrian nonfatal crashes. Due to the fact that minor pedestrian-involved crashes are often resolved privately, the proportion of fatal crashes in Beijing’s data is relatively high.

The pedestrian-involved crash data include time of day, location, pedestrian and vehicle attributes, part of the infrastructure, and environmental and temporal attributes. Further, information including pavement, intersection or not, visibility, and road surface condition is collected by multi-sensors from police, including laser range finders and laser scanning devices. Information, including weather, crash hour, and Workday or not, is supplemented through comparisons with weather databases and holiday databases. Factors such as functional zone and road type are added by comparisons with geographic information systems and other devices. Hence, collecting from several sensor-based sources and other relative sources, the influencing factors are constructed from pedestrian and vehicle attributes, infrastructure attributes, and environmental and temporal attributes. Table 1 presents the summary statistics of the variables.

## 3. Methodology

### 3.1. Random Parameter Logit Model

The RPL model has been widely applied to study crash injury severity, addressing unobserved heterogeneity by permitting parameter estimates to fluctuate across different crash observations. The random utility function for crash *n* in injury severity category *i* is expressed as [8]

(1) $$S_{in}=\beta_{i}X_{in}+\varepsilon_{in}$$

In this case,$X_{in}$ refers to a vector of explanatory variables, $\beta_{i}$ denotes a vector of parameters to be estimated that correspond to $X_{in}$, and $\varepsilon_{in}$ represents a random error term. Accounting for the variability in these parameters, the probability of an outcome in the RPL model for injury severity can be expressed as

(2) $$Pn(i)=\int \frac{\exp(\beta_{i}X_{in})}{\sum \exp(\beta_{i}X_{in})}f(\beta \left|\varphi \right.)d\beta$$

For the purpose of accounting for the variation in the mean of the random parameter, *β_in_* is regarded as a vector of estimable parameters varying in a crash, which is defined as

(3) $$\beta_{in}=\beta +\Theta_{in}Z_{in}+\sigma_{in}\exp(\omega_{in}W_{in})v_{in}$$

where β is the mean parameter estimate across all observations; $Z_{in}$ represents a vector of attributes that captures variations in the mean, with $\Theta_{in}$ being the associated parameter vector; $W_{in}$ denotes a vector of attributes that accounts for variability in the standard deviation $\sigma_{in}$, with its corresponding parameter vector $\omega_{in}$; and $v_{in}$ is a distributed term.

### 3.2. Random Forest

Random forest (RF) represents an advanced ensemble learning technique built upon the principles of decision trees. It involves generating multiple subsets of the original data through bootstrap resampling and constructing a separate decision tree for each subset [20]. In RF, each decision tree is developed independently and incorporates randomness, ensuring a diverse set of predictors. The algorithm applies a bagging method, where repeated random sampling from the training data is used to train individual decision trees.

Given X as the feature set {x_1_, x_2_, …, x_n_} and Y as the target set {y_1_, y_2_, ⋯, y_n_}, and *i* = 1, 2, ⋯, I, the procedure for creating a random forest can be outlined: (1) draw a random subset from {X, Y}, represented as {x*_i_*, y*_i_*}; (2) construct a decision tree *f_i_* using this subset {x*_i_*, y*_i_*}; (3) perform steps 1 and 2 for I times to obtain I decision trees {f_1_, f_2_, ⋯, f_I_}; and (4) combine the predictions from all I decision trees for a given input $\hat{x}$ to form the ensemble prediction function $\hat{f}$ of the random forest.

One of the benefits of RF over similar ML algorithms is that it is less costly and requires fewer hyperparameters. Three fundamental parameters must be specified before training a random forest model: *n* tree (number of trees), *m* try (number of features suitable for splitting), and node size (minimum size of terminal nodes). These parameters play a crucial role in determining the model’s final predictive performance. For hyperparameter tuning, both grid search and Bayesian hyperparameter search are currently common methods.

### 3.3. Shapley Additive exPlanations (SHAP)

As machine learning models grow increasingly sophisticated with enhanced computational capabilities, their internal mechanisms and decision-making processes become opaquer. While achieving high prediction accuracy is important, it is not sufficient to establish a model’s credibility. To address this issue, enhancing the interpretability of these “black box” models is essential for ensuring their generalizability and reliability in practical applications. In 2017, Lundberg et al. [21] introduced SHAP (SHapley Additive exPlanations), a widely adopted technique based on cooperative game theory (CGT). SHAP provides a framework for explaining the predictions of various models, including complex and less transparent ones, by elucidating the reasons behind both classification and regression outcomes [18].

SHAP quantifies the impact of each feature on the model’s prediction by representing the prediction as the sum of the Shapley values associated with each input feature. This approach offers an in-depth explanation of how individual features contribute affect the final prediction:

(4) $$g(x^{\prime })=\varphi_{0}+\sum_{M}^{j=1}\varphi_{j}$$

where *g*(*x*′) denotes the model’s output value, $\varphi_{0}$ indicates the baseline value (i.e., the predicted mean of all training samples), and $\varphi_{j}$ denotes the Shapley value for each feature. SHAP is considered superior to other methods not only because it ensures a consistent interpretation of a model’s predictions but also because it provides detailed insights into the local importance of each feature.

## 4. Results and Discussion

### 4.1. Results of the Random Parameter Logit Model

The results of RPL model are presented in Table 2. The values of AIC and McFadden Pseudo R-squared indicate an acceptable model goodness-of-fit.

(1) Pedestrian and vehicle attributes

The variable gender of pedestrians is significant, with males increasing the likelihood of fatal crashes compared with females. The possible reason may be related to behavioral differences and psychological factors in male pedestrians. For example, male pedestrians may engage in riskier behaviors when walking or crossing streets, such as walking faster, ignoring pedestrian signals, or crossing in unsafe locations. In addition, males may exhibit higher levels of confidence and may underestimate the risks associated with crossing streets. Additionally, the gender of pedestrians is identified as a random parameter, which indicates that for 42.86% of the observations, female pedestrians increase the likelihood of fatal crashes.

The variable motor vehicle type is found to be randomly distributed with a mean of 1.868 and a variance of 3.662, which indicates that motorcycles reduce the likelihood of fatal crashes compared with passenger cars for 69.5% of the observations and increase the likelihood of being fatal for 30.5% of the observations. In addition, possible heterogeneity in means is found. For crashes involving motorcycles, crashes involving female pedestrian increases the mean, which leads to the increased likelihood of fatal injuries.

In terms of motor vehicle license ownership, vehicles with license plates from other places are more likely to be involved in fatal crashes. One possible explanation for this could be that drivers with Beijing license plates are more familiar with the city’s traffic conditions and can better avoid fatal crashes.

Finally, in relation to crash type, side impact is found to have an increased likelihood of severe crashes compared with front impact. This may be due to the fact that during a front impact, drivers have better visibility and can take evasive actions, such as slowing down, thereby reducing accident severity. However, during a side impact, it is much harder for drivers to see pedestrians.

(2) Infrastructure attributes

In the variable of functional zones, urban areas increase the probability of fatal crashes compared with suburban areas. This may be attributed to higher pedestrian flow and more complex traffic conditions in urban areas, which make fatal crashes more likely.

In terms of pavement type, other types of pavement are linked to a higher probability of fatal crashes compared with asphalt. One possible explanation for this is that asphalt pavement offers better traffic conditions and enables vehicles to decelerate more effectively, thereby producing a lower likelihood of being fatal.

(3) Environmental and temporal attributes

The variable lighting condition plays a significant role, as dark–unlighted conditions increase the probability of fatal crashes compared with daytime, which is in line with previous research [22]. Additionally, the lighting condition produces a random parameter. This source of heterogeneity may be related to the endogeneity of this factor [23].

In terms of the variable season, spring increases the likelihood of fatal crashes compared with autumn. This indicates that management authorities should pay particular attention to spring and consider proposing targeted pedestrian accident prevention measures.

### 4.2. Results of the Random Forest

**Figure 1:**
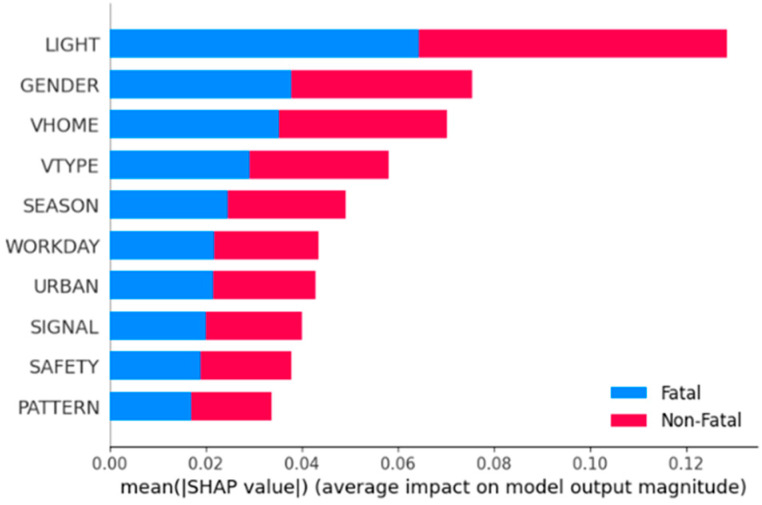
SHAP value bar plot.

The ranking factors of pedestrian injury severity based on RF-SHAP are shown in Figure 1. This study selects the top 10 factors as the most important influence factors.

**Figure 2:**
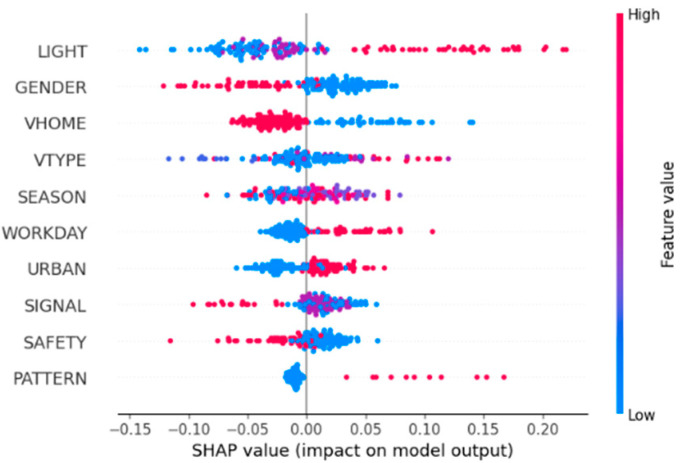
SHAP value summary plot.

Further analysis is conducted on the effect of these ten factors on injury severity. Figure 2 shows the influence, both positive and negative, on the values of injury severity. It can be observed that the importance rank of the factors is lighting condition, gender of pedestrian, motor vehicle license ownership, motor vehicle type, season, Workday or not, functional zone, signal control mode, crash type, and road safety attribute.

**Figure 3:**
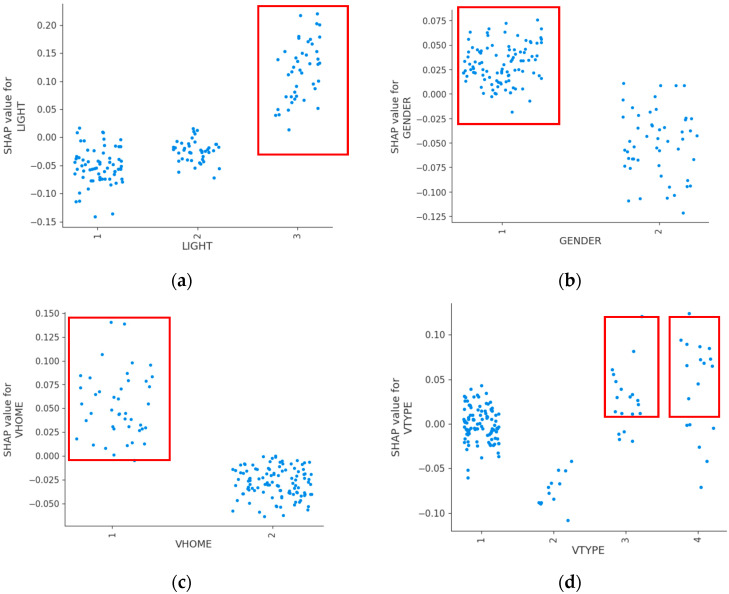

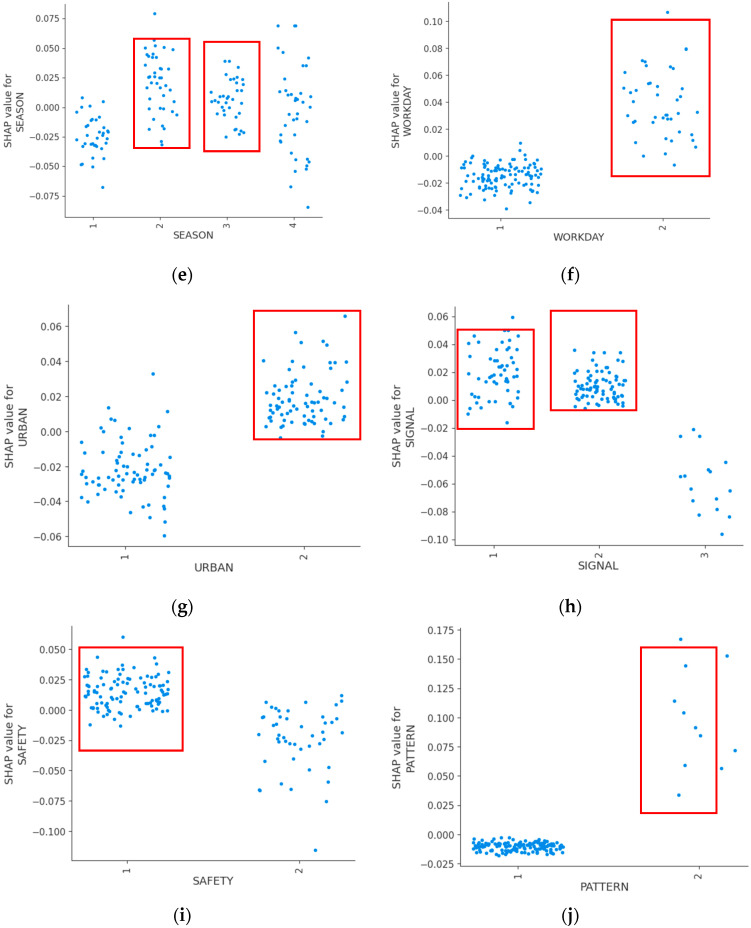
Individual effects of important features (top ten): (**a**) lighting condition, (**b**) gender of pedestrian, (**c**) motor vehicle license ownership, (**d**) motor vehicle type, (**e**) season, (**f**) Workday or not, (**g**) functional zone, (**h**) signal control mode, (**i**) road safety attribute, (**j**) crash type.

Figure 3 shows the details of the feature contributions of the top ten features. The results indicate that darkness–unlighted, male, non-Beijing motor vehicle license, truck and bus, summer and autumn, weekend, suburban, No signal and Other security facilities, normal road, and front impact crashes produce a higher probability of fatal crash occurrences.

### 4.3. Additional Insights Based on the Hybrid Method

Considering that the RPL model can uncover the significant factors and unobserved heterogeneity, and the RF-SHAP can identify important influencing factors and feature contributions, the consistency and complementarity of the results need to be further discussed in order to obtain more effects on pedestrian injury severity. The summary of factors with a high probability of injury severity is represented in Table 3.

(1) Method Consistency

Most conclusions from both methods are in agreement. Key factors to focus on include the gender of the pedestrian (male), motor vehicle type (passenger car), motor vehicle license ownership (others), functional zone (suburban), pavement (others), and lighting condition (darkness–unlighted).

(2) Complementarity

While the results of the RPL model suggest that autumn increases the likelihood of fatal crashes, the RF-SHAP method further reveals that summer also has a higher probability of fatal crashes. Factors such as Workday or not (not), Signal control mode (No signal and Other security facilities), and Road safety attribute (normal road) are not significant according to the RPL model. However, these factors are identified as important in the RF-SHAP analysis. Relying solely on the RPL model may overlook these factors.

Similarly, while crash type (side impact) is not identified as an important factor in the RF results, it is a significant factor in the RPL model. Therefore, using only the RF model would also neglect this factor.

### 4.4. Policy Implications Based on the Hybrid Approach

Based on the results of the hybrid approach, some essential suggestions and policies for pedestrian safety should be implemented.

First, both methods suggest that male pedestrians have been found to be the contributing factors to severe crash outcomes. In fact, many researchers have concluded that the gender of a pedestrian affects the severity of crashes [24]. Jarvis et al. argued that men and women experience the city differently and urged for consideration of gender influences in transportation and urban studies [25]. Hence, consideration of gender impact in urban planning, transport operations, and safety should be strengthened, especially in the planning of pedestrian environments. In addition, the hybrid approach shows that crashes that occur in darkness–unlighted in suburban zones often lead to severe outcomes. The factor “lighting conditions” has been shown to be an important factor affecting the injury severity and frequency of crashes in different types of traffic accidents. Hence, lighting conditions are an important environmental factor in road safety research. Moreover, campaigns and awareness programs designed for rural areas should emphasize walking against traffic and using safety equipment, such as wearing reflective clothing, using lights, etc.

Additionally, the hybrid approach indicates that crashes occurring at night in suburban areas often result in severe outcomes. Therefore, it is essential to install additional lighting facilities in areas with high pedestrian crash rates in suburban areas. Moreover, campaigns and awareness programs targeted at suburban areas should emphasize the importance of walking against traffic and utilizing safety equipment, such as wearing reflective clothing and using lights. The hybrid approach also indicates that vehicles with license plates from other places are more likely to be involved in fatal crashes than those from Beijing. This conclusion shows that special attention should be paid in Beijing to the safety education of the users of vehicles with nonlocal license plates in the management of pedestrian safety.

Finally, the RF-SHAP model shows that No signal and Other security facilities increase the probability of pedestrian fatality in a pedestrian–vehicle crash. Hence, several ITS-related measures, such as installing an adaptive signal control system, can be proposed at intersections to promote pedestrian crossing safety.

## 5. Conclusions

This paper focuses on the influencing factors of pedestrian injury severity using sensor-based data amidst the background of the rapid development of ITS. It proposes a hybrid approach of the RPL model and RF-SHAP. By utilizing multi-source data, including police-reported pedestrian crash data and relevant data from other sources in Beijing, this study identifies eight significant influencing factors, with seven of them being deemed important according to the random forest analysis. Additionally, factors such as Workday or not (Not), Signal control mode (No signal and Other security facilities), and Road safety attribute (Normal road) are not significant in the RPL model but are identified as important in the RF analysis. Focusing solely on significant or important factors may lead to the omission of some conclusions, highlighting the significance of this hybrid approach.

In summary, several ITS-related measures can be proposed to mitigate pedestrian fatal crashes. For example, targeted education for male pedestrians to emphasize adherence to traffic rules; issuing reminders to drivers with non-Beijing motor vehicle licenses when they obtain entry permits, advising them to be cautious of pedestrians; and ensuring that motor vehicles exercise extra caution in specific environments, such as suburban areas, roads with other types of pavements, darkness–unlighted conditions, and intersections with no signal or other security facilities.

However, this study has certain limitations. First, different hybrid methods between statistical models and machine learning approaches should be investigated for pedestrian safety research to uncover additional insights. Second, more sensor-based data should be collected for further study.

## Figures and Tables

**Table 1:** Description of pedestrian–vehicle crash data.

Variable	Description	Proportion
*Pedestrian and vehicle attributes*		
Gender of pedestrian (GENDER)	1 = Male	64.68%
	2 = Female	35.32%
Motor vehicle type (VTYPE)	1 = Passenger car	63.36%
	2 = Motorcycle	8.47%
	3 = Truck	25.66%
	4 = Bus	2.51%
Motor vehicle license ownership (VHOME)	1 = Others	31.08%
	2 = Beijing	68.92%
Crash type (PATTERN)	1 = Side impact	92.46%
	2 = Front impact	7.54%
***Infrastructure attributes***		
Functional zone (URBAN)	1 = Urban	46.43%
	2 = Suburban	53.57%
Lane group (LGROUP)	1 = Motor vehicle lane	67.06%
	2 = Others	32.94%
Physical isolation facility (ISOLATION)	1 = No isolation	39.55%
	2 = Isolation	60.45%
Pavement (PAVEMENT)	1 = Asphalt	97.22%
	2 = Others	2.78%
Intersection or not (INTERSECTION)	1 = Intersection	18.92%
	2 = Road section	81.08%
Road type (RTYPE)	1 = High-grade highway	17.46%
	2 = Low-grade highway	8.73%
	3 = Expressway	8.47%
	4 = Trunk road	33.99%
	5 = Other road	31.35%
Signal control mode (SIGNAL)	1 = No signal	32.54%
	2 = Other security facilities	55.95%
	3 = Signal	11.51%
***Environmental and temporal attributes***		
Weather (WEATHER)	1 = Sunny and cloudy	84.92%
	2 = Severe weather	15.08%
Lighting condition (LIGHT)	1 = Daytime	43.65%
	2 = Darkness–lighted	32.67%
	3 = Darkness–unlighted	23.68%
Visibility (VISIBILITY)	1 < 50 m	10.45%
	2 = 50–100 m	25.79%
	3 > 100 m	63.76%
Road surface condition (SURFACE)	1 = Dry	91.53%
	2 = Others	8.47%
Road safety attribute (SAFETY)	1 = Normal road	64.95%
	2 = Section with lurking peril	35.05%
Crash hour (CH)	1 = Rush hour	20.11%
	2 = Others	79.89%
Workday or not (WORKDAY)	1 = Yes	71.96%
	2 = Not	28.04%
Season (SEASON)	1 = Spring	26.32%
	2 = Summer	23.28%
	3 = Autumn	26.59%
	4 = Winter	23.81%

**Table 2:** Model estimation and results.

Factor	Coefficient	Z-Stat	Marginal Effects
Constant	−0.125	−0.87	
***Pedestrian and vehicle attributes***			
Gender of pedestrian (base: Female)			
Male	0.443	3.19	0.110
*Standard deviation*	2.437	11.03	
Motor vehicle type (base: Passenger car)			
Motorcycle	−1.868	−3.92	−0.463
*Standard deviation*	3.662	4.38	
Motor vehicle license ownership (base: Beijing)			
Others	0.420	2.79	0.104
Crash type (base: Front impact)			
Side impact	1.171	3.67	0.290
***Infrastructure attributes***			
Functional zone (base: Suburban)			
Urban	−0.415	−2.82	−0.103
Pavement (base: Asphalt)			
Others	1.324	2.37	0.328
***Environmental and temporal attributes***			
Lighting condition (base: Daytime)			
Darkness–unlighted	0.842	4.43	0.209
Standard deviation	0.929	3.91	
Season (base: Autumn)			
Spring	−0.301	−1.89	−0.075
**Heterogeneity in means of random parameters**			
Motor vehicle type (Motorcycle): Gender of pedestrian (Female)	2.861	2.51	
**Goodness-of-fit measures**			
AIC	975.9		
Log-likelihood at zero	−657.857		
Log likelihood function	−474.973		
McFadden Pseudo R-squared	0.278		

**Table 3:** Factors with high probability of injury severity.

RPL	RF
Gender of pedestrian (Male)	Gender of pedestrian (Male)
Motor vehicle type (Passenger car)	Motor vehicle type (Passenger car)
Season (Autumn)	Season (Summer and Autumn)
Functional zone (Suburban)	Functional zone (Suburban)
Pavement (Others)	Pavement (Others)
Motor vehicle license ownership (Others)	Motor vehicle license ownership (Others)
Lighting condition (Darkness–unlighted)	Lighting condition (Darkness–unlighted)
Crash type (Side impact)	
	Workday or not (Not)
	Signal control mode (No signal and Other security facilities)
	Road safety attribute (Normal road)

## Data Availability

Dataset available on request from the authors.
